# Is social function a good proxy measure of personality disorder?

**DOI:** 10.1002/pmh.1513

**Published:** 2021-05-17

**Authors:** Peter Tyrer, Min Yang, Helen Tyrer, Mike Crawford

**Affiliations:** ^1^ Centre for Psychiatry Imperial College London London UK; ^2^ School of Public Health West China Medical Center of Sichuan University Chengdu China; ^3^ Faculty of Health, Art and Design Swinburne University of Technology Melbourne Australia

## Abstract

**Background:**

Personality assessment may be helped by proxy measures.

**Aims:**

To examine the assessment of social functioning in relationship to personality disorder.

**Method:**

Secondary analysis of data from three clinical studies, following deliberate self‐harm (*n* = 460), cognitive behaviour therapy for health anxiety (*n* = 444) and a 30‐year follow‐up of 200 anxious/depressed patients. Social function and personality were assessed using the Social Functioning Questionnaire (SFQ) and the Personality Assessment Schedule, with its ICD‐11 modification. A 5‐item short version of the SFQ, the Short Social Functioning Questionnaire (SSFQ), was also developed.

The SFQ score in the first two studies (area under curve [AUC] 0.64 and 0.65) partly predicted personality status; in the third study, this achieved close agreement (AUC SFQ 0.85 [95% CI 0.8–0.9]; AUC SSFQ 0.84 [95% CI 0.78–0.89]). In all studies, social function deteriorated linearly with increasing personality pathology. Cut‐off points of 4 on the SSFQ and 7 on the SFQ had high sensitivity (SSFQ 82%–90%; SFQ 82%–83%) and acceptable specificity (SSFQ 66%–75%; SFQ 69%–75%) in identifying personality disorder in the third study.

**Conclusions:**

Social functioning recorded in either a 5‐item or 8‐item self‐rating is a useful proxy measure of personality disturbance and may be the core of disorder.

## INTRODUCTION

The assessment of personality disorder is not currently an easy process. Very few of the comprehensive instruments available can be completed in less than an hour. The two standard instruments for assessing personality disorder, the SCID‐5‐PD (First et al., 2016) and the International Personality Disorder Examination (IPDE) (Loranger et al., 1994), can take up several hours to complete. Even established self‐rated instruments are complex, the widely used Personality Inventory for DSM‐5 (PID‐5) has the same number of questions (220) in both self‐report and informant versions, and this does not deliver an assessment of full personality disorder (Krueger et al., 2012).

As a consequence of this concern, various screening instruments have been introduced, including the Standardized Assessment of Personality—Abbreviated Scale (SAPAS) (Moran et al., 2003), the Iowa Personality Disorder Screen (IPDS) (Langbehn et al., 1999), the Standardized Assessment of Severity of Personality Disorder (SASPD) (Olajide et al., 2018), the Levels of Personality Functioning Scale—Brief Form (LPFS‐BF) (Weekers et al., 2019) and the Personality Assessment Questionnaire for ICD‐11 (Kim et al., 2021). These are much shorter, and most only take a few minutes to complete.

It is now considered an advantage to have personality status assessed on every patient being assessed in psychiatric practice. In many situations, screening questionnaires such as those mentioned above are being used, but most are more suitable for excluding personality disorder than in detecting it. There is also still a level of stigma in using assessments of personality that may affect response decisions; this is less likely to happen with measures of social function.

The ICD‐11 classification of personality disorder allows all levels of personality dysfunction to be recorded, including that of personality difficulty, a sub‐syndromal condition (Tyrer et al., 2019). This is associated with impaired functioning and distress and may be an important component of morbidity in other disorders. It also highlights interpersonal social dysfunction as a key element of impairment, as does the AMPD DSM‐5 model in its requirement for Criterion A. As all mental and physical disorders have an impact on functioning, it might be thought that personality would only play a major part in this. But because in several studies there has been a high correlation between social function and personality status (Carp, 1985; Seivewright et al., 2004; Tse et al., 2011), the specificity of the association was felt worthy of further study. In particular, the hypothesis that a broad measure of social function is indicative of personality dysfunction per se without any further assessment was being tested.

In this study, only one measure of social function was studied, the Social Functioning Questionnaire (SFQ) (Tyrer et al., 2005). This is a self‐rating scale of eight items with a maximum score of 24. But because the questions in this scale overlap with many others, including the Social Adjustment Self‐Report Scale (Weissman & Bothwell, 1976) and the Work and Social Adjustment Scale (Mundt et al., 2002), the findings with the SFQ may extend to other scales also.

## METHOD

We conducted a secondary analysis of data from three clinical studies, all of which recorded social function with the SFQ (Tyrer et al., 2005) and personality with the Personality Assessment Schedule (PAS), with two of these involving the shortened version, the Quick Personality Assessment Schedule (PAS‐Q). Because the PAS shows close similarities with the ICD‐11 classification of personality disorder, a harmonized synthesis of the two methods (Tyrer, Cooper, et al., 2014a) allowed the five levels of the ICD diagnostic system also to be recorded.

### Study 1

In this study, the POPMACT study, patients were seen and assessed immediately after a self‐harming episode (Tyrer et al., 2003). The study was a randomized trial of cognitive behaviour therapy (CBT) versus treatment as usual in which assessments of personality status and social functioning were made at baseline using the PAS‐Q and SFQ and further SFQ assessments made at 6 and 12 months. In this study, the expectation was that as function was assessed immediately after a major event that was bound to impact social function, there would be a poor relationship between social function and personality status.

### Study 2

The CHAMP study assessed social function and personality using the SFQ and PAS‐Q in medical patients who had been recruited to a trial of adapted CBT for health anxiety. In this study, the patients were not recruited at a time of crisis but only if their scores on a health anxiety inventory reached a pathological level (Tyrer, Crawford, et al., 2014b). In this study, because it was made at a time of relative lifetime stability, the expectation was that the relationship between the SFQ and PAS‐Q would be stronger. In this study, the individual scores on the eight SFQ items at baseline were assessed for the strength of their relationship with personality. From this study, the analysis of the individual items on the SFQ was studied in relation to personality, and those that were least related were removed. The Short Social Functioning Questionnaire (SSFQ) was created for the other items.

### Study 3

In the third study, the Nottingham Study of Neurotic Disorder (Tyrer et al., 1988), patients were seen at baseline and followed up over 30 years. Personality was assessed using the full PAS at baseline, 2, 12 and 30 years, but the SFQ was given only at 12 and 30 years. In this study, the relationship between personality status and social function was assessed in three ways:

- baseline personality status and social function at 12 years,
- personality status and social function, both at 12 years, for the SFQ and the SSFQ,
- personality status and social function, both at 30 years, for the SFQ and SSFQ.

In all the studies, personality status was measured by versions of the PAS (Tyrer et al., 1979; Tyrer & Alexander, 1979) and its shorter version, the PAS‐Q (Tyrer, 2000). These assess premorbid personality, not current function, and, unlike most personality assessment measures, do not vary with changes in official classifications as they are linked mainly to severity of disturbance (Tyrer, 1988; Tyrer et al., 1990), although algorithms to realign with other classifications exist. The PAS consists of 24 sets of questions about personality attributes, each scored on an 8‐point scale; the PAS‐Q follows the same principle but is shorter, and if screening questions do not reach the threshold for scoring, those sections are omitted (Tyrer, 2000). Both versions score severity of personality disturbance on a 5‐point scale: 0 (*no personality disturbance*), 1 (*personality difficulty*), 2 (*simple personality disorder* [subsequently renamed ‘mild personality disorder’ in ICD‐11]), 3 (*diffuse personality disorder* [subsequently named ‘moderate personality disorder’ in ICD‐11]) and 4 (*severe personality disorder*) (as in ICD‐11) (Tyrer et al., 2019).

Ethical approval for all three studies was granted from the London Multicentre Research Ethics Committee (POPMACT), Nottingham Research Ethics Committee (CHAMP) and Northampton Research Ethics Committee (Nottingham Study).

## PLAN OF ANALYSIS AND RESULTS

### Step 1

As all mental health disturbance affects social function, the expectation was that in Studies 1 and 2, there would be additional elements of impairment after self‐harm and attendance with anxiety at medical clinics. In Study 3, when assessment was determined by the calendar and not by events, the independence of social function was felt to be stronger. The analysis was therefore carried out first with examination of the relationship between each of the individual items of the SFQ with the personality dysfunction positive, both at 12‐ and 30‐year follow‐up, their relationship with the PAS total score, and also relationship with individual personality disorder. Spearman and Pearson correlation analysis was performed.

The results in Table 1 suggested that there was highly significant agreement (44 out of 48 correlation coefficients had *p*‐values smaller than 0.001) between the SFQ scores and the presence of personality disorder by ICD‐11 and PAS definitions, with five items of the SFQ (1,2, 4, 7 and 8) showing higher correlations than others. The SSFQ was created with these five items (Appendix A).

**TABLE 1:** Correlations between social function items in the Social Functioning Questionnaire (SFQ) and personality diagnosis (ICD‐11) and total scores on the Personality Assessment Schedule (PAS) (significance *p*‐value in brackets) in Study 3

	DSM IV PD positive		ICD‐11 PD positive		PAS total score	
	Spearman correlation coefficient		Spearman correlation coefficient		Pearson correlation coefficient	
SFQ items	12 years	30 years	12 years	30 years	12 years	30 years
	*n* = 185	*n* = 88	*n* = 185	*n* = 88	*n* = 185	*n* = 88
1^a^	0.381 (0.000)	0.494 (0.000)	0.332 (0.000)	0.530 (0.000)	0.451 (0.000)	0.404 (0.000)
2^a^	0.391 (0.000)	0.388 (0.000)	0.366 (0.000)	0.546 (0.000)	0.523 (0.000)	0.509 (0.000)
3	0.291 (0.000)	0.230 (0.031)	0.327 (0.000)	0.379 (0.000)	0.389 (0.000)	0.404 (0.000)
4^a^	0.416 (0.000)	0.515 (0.000)	0.377 (0.000)	0.555 (0.000)	0.515 (0.000)	0.414 (0.000)
5	0.331 (0.000)	0.174 (0.105)	0.277 (0.000)	0.215 (0.045)	0.372 (0.000)	0.282 (0.007)
6	0.322 (0.000)	0.320 (0.002)	0.272 (0.000)	0.404 (0.000)	0.357 (0.000)	0.340 (0.001)
7^a^	0.525 (0.000)	0.486 (0.000)	0.527 (0.000)	0.545 (0.000)	0.615 (0.000)	0.552 (0.000)
8^a^	0.531 (0.000)	0.437 (0.000)	0.517 (0.000)	0.598 (0.000)	0.562 (0.000)	0.472 (0.000)

^a^ Items with higher correlations included in the SSFQ.

The items in the SSFQ were also better predictors of individual personality disorders in Study 3 with the exception of those initially diagnosed as sociopathic in the original classification (Tyrer & Alexander, 1979) (Table 2). This is largely explained by the greater improvement in those with antisocial personality features in that cohort over the course of time (Seivewright et al., 2002).

**TABLE 2:** Pearson's R between score level of SFQ items and ICD‐11 personality disorder status of specific type in Study 3

PD type	SFQ 1	SFQ 2	SFQ 3	SFQ 4	SFQ 5	SFQ 6	SFQ 7	SFQ 8
12 years (*n* = 185)								
Sociopathic	0.183*	0.097	0.203**	0.239**	0.300***	0.151*	0.176*	0.167*
Passive dependent	0.340***	0.278***	0.281***	0.265***	0.180*	0.198**	0.291***	0.315***
Anankastic	0.184*	0.290***	0.142	0.232**	0.239**	0.218**	0.320***	0.318***
Schizoid	0.380***	0.246**	0.290***	0.440***	0.279***	0.283***	0.427***	0.376***
30 years (*n* = 88)								
Sociopathic	0.189	0.023	0.231*	0.217*	0.194	0.333**	0.195	0.141
Passive dependent	0.395***	0.304**	0.166	0.278**	0.089	0.301**	0.501***	0.405***
Anankastic	0.311**	0.261*	0.085	0.410***	0.118	0.228*	0.414***	0.340**
Schizoid	0.284**	0.278**	0.168	0.540***	0.076	0.210*	0.319**	0.270*

* *p* < 0.05.

** *p* < 0.01.

*** *p* < 0.001.

### Step 2

Further analysis, using the data in Study 3 with both the SFQ and SSFQ, examined dose–response effects between the severity level of personality disorder and mean scores of the SFQ and SSFQ by means of the *χ* ^2^ test for difference between severity levels and linear trend by severity level of personality disorder.

The results (Table 3) showed that higher scores on the SFQ and SSFQ were strongly associated with the severity level of personality disorder (*p*‐values between 0.028 and 0.000 for difference), with a graded increase in both social function scores with increasing severity (*p*‐values between 0.029 and 0.000 for linearity). More severe personality disorder (moderate) scored 90%–224% higher on the SFQ and 224%–268% higher on the SSFQ than those with no personality dysfunction. This relationship was still strong when comparing social function with baseline personality status. Those with moderate or severe personality disorder at baseline had scores 73%–86% higher on the SFQ and 76%–81% higher on SSFQ at 12‐ and 30‐year follow‐up, respectively.

**TABLE 3:** Mean scores of SFQ and SSFQ by ICD‐11 personality level in Study 3

	12 years (*n* = 178–185)			30 years (*n* = 86–88)		
Personality status	*n*(%)	SFQ mean (SD)	SSFQ mean (SD)	*n*(%)	SFQ mean (SD)	SSFQ mean (SD)
*PD status by follow‐up year*						
All		7.77(5.38)	4.84(3.85)		7.92(5.56)	5.25(4.01)
Any PD(+)^a^	77(41.6)	11.19(5.13)	7.23(3.66)	41(46.6)	12.00(4.64)	8.22(3.37)
Any PD(−)	108(58.4)	5.32(4.08)	3.13(2.98)	47(53.4)	4.36(3.45)	2.66(2.42)
P value for difference AUC value (SE)		0.000 0.815(0.031)	0.000 0.810(0.032)		0.000 0.895(0.033)	0.005 0.898(0.035)
*PD status of follow‐up year*						
No PD	77(41.6)	6.14(4.43)	2.39(2.49)	29(33.0)	4.00(3.96)	2.41(2.75)
PD difficulty	31(16.7)	7.55(4.21)	4.97(3.33)	18(20.5)	4.94(2.41)	3.06(1.76)
PD mild	23(12.4)	8.00(4.12)	4.78(2.66)	18(20.5)	10.61(5.20)	7.17(3.93)
PD moderate	40(21.6)	11.67(4.61)	7.75(3.39)	22(25.0)	12.95(3.96)	8.86(2.57)
PD severe	14(7.6)	15.07(5.15)	9.79(3.64)	1(1.1)	16.00(n/a)	13.00(n/a)
*p*‐Value for difference		0.000	0.000		0.000	0.000
*p*‐Value for linearity		0.000	0.000		0.000	0.000
*The baseline PD status*						
No PD	75(42.1)	6.27(4.42)	3.87(3.32)	35(40.7)	6.29(4.59)	4.29(3.44)
PD difficulty	39(21.9)	7.82(5.58)	4.85(3.63)	15(17.4)	8.87(6.28)	6.33(4.39)
PD mild	43(24.2)	9.56(5.70)	6.09(4.16)	24(27.9)	8.17(5.78)	5.08(4.32)
PD moderate/severe	21(13.8)	10.86(5.51)	6.81(4.31)	12(14.0)	11.67(5.40)	7.75(3.49)
*p*‐Value for difference		0.000	0.002		0.028	0.047
*p*‐Value for linearity		0.000	0.000		0.007	0.029

^a^ Defined as PD mild or moderate or severe.

### Step 3

The same method used in Step 2 was used to examine the data in the other two studies. In Study 1, the individual scores for the SFQ were not recorded in the database, so an SSFQ could not be created. The findings of the total scores indicated a significant dose–response relationship (*p* < 0.0001) between severity of personality disturbance and total SFQ score (Table 4). This was maintained after assessments at 6 and 12 months (*p* < 0.0001).

**TABLE 4:** Social functioning questionnaire (mean score) at each level of personality disturbance in the POPMACT sample (Study 1)

	Baseline (*n* = 477)	6 months (*n* = 388)	12 months (*n* = 400)
Baseline personality status	*n*(%): mean (SD)	*n*(%): mean (SD)	*n*(%): mean (SD)
No PD symptoms	43(9.0): 11.19(3.95)	37(9.5): 9.03(4.43)	36(9.0): 8.94(4.52)
PD difficulty	234(49.1):12.39(4.58)	186(47.9):9.81(4.94)	193(48.3): 9.30(5.05)
PD simple (mild) disorder	124(26.0):14.32(4.19)	108(27.8):11.10(4.52)	107(26.8):10.36(5.05)
PD diffuse (moderate) disorder	76(15.9): 15.51(4.25)	57(14.7): 13.32(4.17)	64(16.0):12.73(5.04)
*p*‐Value for difference	0.000	0.000	0.000
*p*‐Value for linearity	0.000	0.000	0.000
All	13.28(4.57)	10.61(4.83)	10.10(5.10)
PD(+)^a^	200(41.9): 14.77(4.25)	165(42.5): 11.86(4.51)	171(42.8): 11.25(5.17)
PD(−)	277(58.1): 12.21(4.50)	223(57.5): 9.68(4.86)	229(57.2): 9.24(4.89)
*p*‐Value^b^	0.00	0.000	0.000
AUC value (SE)	0.651 (0.025)	0.637 (0.028)	0.614 (0.028)

^a^ Defined as mild or moderate PD.

^b^ For difference in the means between PD(+) and PD(−) groups.

In Study 2 (CHAMP), the individual SFQ scores were recorded, and so, scores for the SFQ and SSFQ could be obtained. Both showed a strong dose–response relationship with personality status (*p* < 0.0001), with those with moderate levels of personality disorder scoring 98%–120% more on the scales than for those with no personality disturbance (Table 5).

**TABLE 5:** Mean scores of long SFQ and short SSFQ scales by level of personality dysfunction in CHAMP sample (Study 2)

	SFQ (*n* = 441)	SSFQ (*n* = 441)
Personality status	*n*(%): mean (SD)	*n*(%): mean (SD)
No personality disturbance	46(10.4): 6.68(3.87)	46(10.4): 4.04(2.72)
Personality difficulty	255(57.8): 9.14(4.31)	255(57.8): 5.87(3.24)
Mild personality disorder	104(23.6): 10.24(4.55)	104(23.6): 6.92(3.20)
Moderate personality disorder	36(8.2): 13.23(3.85)	36(8.2): 9.06(2.96)
*p*‐Value for difference	0.000	0.000
*p*‐Value for linearity	0.000	0.000
All	9.48(4.52)	6.19(3.35)
PD(+)^a^	140(31.7): 11.01(4.56)	140(31.7): 7.47(3.26)
PD(−)	301(68.3): 8.76(4.33)	301(68.3): 5.59(3.23)
*p*‐Value^b^	0.000	0.000
AUC value (SE)	0.638 (0.028)	0.661(0.027)

^a^ Defined as mild or moderate PD.

^b^ For differences in the means between PD(+) (personality disorder) and PD(−) (no personality dysfunction and personality difficulty groups).

### Step 4

**FIGURE 1:**
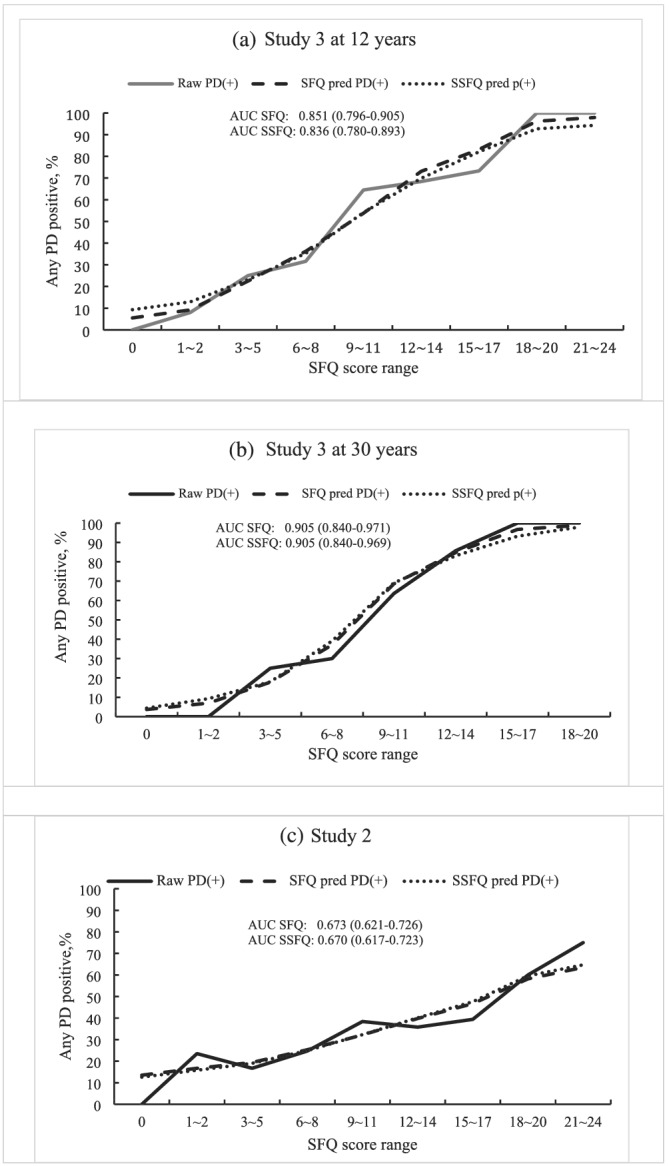
Probability of positive diagnosis of personality disorder over the social function score range—comparison between the observed and model predicted positive rates in Studies 2 and 3

This step of the analysis examined the predictive accuracy of the SFQ and the SSFQ items, based on logistic regression models that included eight or five items, respectively, in the two scales, for any personality disorder using Studies 2 and 3. The model estimated the probability of a positive diagnosis of personality disorder at different ranges of SFQ scores and when compared with the actual rate of observed positive disorder cases in the same score range. Overall accuracy measure, the area under curve (AUC) value, as well as their 95% confidence intervals was calculated for the predicted probability of personality disorder (Figure 1). The results showed little difference between the SFQ and the SSFQ in their ability to detect any personality disorders in the two studies, but much higher predictive accuracy for individuals in Study 3 with AUC values as 0.851 (95%CI: 0.796–0.905) for SFQ and 0.836 (95%CI: 0.780–0.893) for SSFQ at 12 years, than those in Study 2, with AUC values as 0.673 (95%CI: 0.621–0.726) for SFQ and 0.670(95%CI: 0.617–0.723) for SSFQ.

### Step 5

The final set of the analysis tried to identify the cut‐off scores for detection of personality disorder. A screening instrument needs to be highly sensitive (i.e. successful in identifying all cases) (few false positives) and yet reasonably specific (i.e. few false negatives). For the Study 3 sample, a score of 4 on the short (SSFQ) questionnaire and one of 7 on the original SFQ had the greatest likelihood of achieving these aims, with the sensitivity of both versions conspicuously high (SFQ 82%–83%; SSFQ at 82%–90%) and specificity at acceptable levels (SFQ 69%–75%; SSFQ at 66%–75%) (Table 6). Taking into account PD prevalence in population, the positive predictive value (PPV) for SFQ at the cut‐off point 7 was between 65.6% and 73.9%, and the negative predictive value (NPV) was between 83.3% and 84.3%. The PPVs and NPVs for SSFQ at the cut‐off point 4 were 63%–75.5% and 83.5%–89.7%, respectively. The higher NPVs than PPVs suggested that using the SFQ score of 7 or SSFQ score of 4 to screen personality dysfunction in the general population would pick up more true negative cases than true positive. Raising the cut‐off score to 9 for SFQ and to 7 for SSFQ could have a balanced PPV and NPV as a screening tool. However, in the Study 1 and Study 2 populations, the cut‐off score was different, at 10 on the SFQ scale and 6 on the SSFQ scale from the Study 2 sample and at about 10 on the SFQ scale from the Study 1 sample. Thus, the social function scores had much lower sensitivity and specificity, that is, lower predictive accuracy overall in latter two samples than that in the Study 3 sample.

**TABLE 6:** Sensitivity and specificity of SFQ/SSFQ score for identification of any personality disorder

	SFQ					SSFQ				
	Cut‐off score	Sen(%)	Spe(%)	PPV(%)	NPV(%)	Cut‐off score	Sen(%)	Spe(%)	PPV(%)	NPV(%)
*Study 3 sample*										
12 years	7	81.8	69.4	65.6	84.3	3	89.6	52.8	57.5	87.7
	8	76.8	75.0	68.8	81.8	4	81.8	65.7	63.0	83.5
	9	68.8	80.6	76.1	78.3	5	76.6	72.2	66.3	81.3
	10	62.3	82.4	71.6	78.4	6	66.2	79.6	69.9	76.8
	11	50.6	87.0	71.6	75.4	7	57.1	83.3	71.0	73.2
30 years	7	82.9	74.5	73.9	83.3	3	92.7	59.6	66.7	90.3
	8	78.0	83.0	80.0	81.3	4	90.2	74.5	75.5	89.7
	9	78.0	87.2	84.2	82.0	5	87.8	78.8	78.3	80.1
	10	73.2	91.5	88.2	79.6	6	78.0	87.2	84.2	82.0
	11	70.7	91.5	87.9	78.2	7	70.7	93.6	90.0	78.6
*Study 2 sample*										
	7	84.3	35.5	37.8	82.9	3	92.9	17.9	34.5	84.4
	8	81.4	43.9	40.3	83.5	4	88.6	29.9	37.0	84.9
	9	71.4	50.2	40.0	79.1	5	83.6	41.2	39.8	84.4
	10	60.7	57.5	39.9	75.9	6	70.7	51.8	40.6	79.2
	11	49.3	64.5	39.2	73.2	7	60.7	64.1	44.0	77.8
*Study 1 sample*										
Baseline	12	75.5	41.5	48.2	70.1		N/A			
	13	68.5	50.2	49.8	68.8					
	14	58.0	57.0	49.4	65.3					
	15	50.5	65.7	51.5	64.8					
6 months	10	70.9	51.6	52.0	70.6					
	11	63.6	60.1	54.1	69.1		N/A			
	12	57.0	65.9	55.3	67.4					
	13	43.6	70.4	52.2	62.8					
12 months	10	64.3	55.5	51.9	67.6					
	11	56.1	62.4	52.7	65.6		N/A			
	12	49.1	68.8	53.8	64.3					

## DISCUSSION

This study has shown reasonably strong correlations between social function scores and personality status in all three populations. It also demonstrated that the short SFQ scale of five items (SSFQ) is very similar to the longer SFQ in predicting personality dysfunction than the full SFQ scale of eight items. However, the predictive accuracy varies among different populations. High and clinically acceptable sensitivity/specificity has been shown by the Nottingham sample, with lower predictive accuracy of SFQ in the other two samples.

The finding of these correlations between social function scores and personality status in all three populations is striking. Social function can be impaired by many other factors apart from personality, and yet, in all these studies, personality disorder comes out very strongly as the strongest indicator of poor social function. In Study 1, the participants all had experienced a self‐harming episode, and many were assessed in hospital (Byford et al., 2003; Tyrer et al., 2003). In Study 2, 77% had an established medical diagnosis at baseline (Tyrer et al., 2020), including diabetes, hypertension and cardiac problems and respiratory disorders such as asthma and chronic obstructive pulmonary disease (COPD), and yet, the effects of these on social function (using the broad parameters of the SFQ) were less than might be expected. Nevertheless, the lower predictive accuracy in the POPMACT and CHAMP studies suggests that when other pathologies are more prominent, the accuracy of diagnosis is reduced. This suggests that the SFQ and SSFQ are better suited to epidemiological studies as screening tools, similar to the situation in the Nottingham Study, when patients were contacted on the 12th and 30th year anniversaries of their randomization.

In a previous study of 100 patients presenting as psychiatric emergencies (Merson et al., 1992), both path and regression analyses were used to assess patients at baseline and over the course of 12 weeks. The results showed that at baseline, both psychopathology and personality pathology contributed to social dysfunction more of less equally, but from 2 weeks onwards, personality abnormality contributed to a greater degree than clinical psychopathology (Nur et al., 2004). Thirty‐five per cent of the patients had a personality disorder, and in these, there was a much stronger correlation between social function scores at baseline and 12 weeks accounting for 48% of the variation, whereas in those with no personality disorder, the correlation was much weaker (14%).

These previous results are in keeping with our findings. In both Studies 1 and 2, there were both mental and physical contributions to social dysfunction, so the mean SFQ scores were between 9.5 and 13.3 (the highest immediately after the episode of self‐harm), whereas in the Nottingham Study, a follow‐up one, the mean scores were lower (7.8 at 12 years and 7.9 at 30 years).

### Implications of findings in understanding personality disorder

The findings, which need to be replicated, particularly with other measures of social function, suggest that other studies, while not purporting to include those with personality disorder, will almost certainly have done so. Thus, Hebert et al. (2011), in a study of 844 subjects attempting to quit smoking, found that 40% had depressive symptoms, with 74% of those with more severe symptoms and 45% of those with milder symptoms having SFQ scores of 10 or more. McCombie et al. (2016), in a randomized study of computerized CBT in inflammatory bowel disease, found mean SFQ scores of 5.8 and 6.7 in their treatment groups, and Lorenz et al. (2018), in a study of the course of adjustment disorder, a condition in which personality disorder might not be expected, found mean SFQ scores of 6.2 of all subjects, with 6.8 for women. These studies suggest that many of these populations may have had personality disorder also. But in this context, the data from studies of specific populations with personality disorder show much higher mean SFQ scores, including Davidson et al. (2006) (mean 13), Huband et al. (2007) (mean 12–14) and McMurran et al. (2017) (mean 13–15), suggesting that in the other clinical populations, the personality disturbance is of a milder degree. In studies of major mental illness such as schizophrenia and bipolar disorder, the relationship with personality disorder may not be the same as impaired social function may be of a different nature in these conditions (Birchwood et al., 1990).

Despite this positive association, it would not be appropriate to regard any of the social functioning questionnaires as substitutes for other tested measures of personality disorder such as the SAPAS (Moran et al., 2003), the currently most used assessment tool. It is also short. The SASPD (Olajide et al., 2018) (despite some criticisms of its psychometric properties; Rek et al., 2020) and the LPFS‐BF (Weekers et al., 2019) are also useful options. Recently, a new scale to record the severity of ICD‐11 personality disorders has also been published (Bach et al., 2021). This has 14 items: four relating to self‐functioning; four to interpersonal functioning; three to cognitive‐perceptual, emotional, and behavioural manifestations, respectively; two to harm; and one related to overall psychosocial impairment. Our findings suggest the last of these might be the most important. But social function is also being measured frequently in other studies, and it is reasonable to suppose that when the level of impairment is much higher than might be expected, personality disorder should be considered. The SFQ and SSFQ might also be used in studies where for any reason it is considered to be inappropriate to have a formal personality measure included.

The format of the ICD‐11 personality diagnosis includes aspects of self‐functioning; aspects of interpersonal functioning and emotional, cognitive, and behavioural manifestations; and a general description of psychosocial functioning (World Health Organization, 2018). All these are covered, at least to some degree, in the SFQ, so may explain the good association with personality disorder.

## CONCLUSIONS

The relationship between social function and personality disorder is a close one that can be summarized:

1. Social function is affected by personality disorder, mental and physical illness.
2. When a recent episode of mental and physical illness is prominent, a significant component of social dysfunction (about 20%–40%) is contributed by these pathologies, the rest from personality dysfunction.
3. In the absence of recent episodes of mental and physical illness, social dysfunction is strongly associated with personality disorder.
4. A score of 4 on the short (SSFQ) questionnaire and one of 7 on the longer SFQ questionnaire may be indicative of personality disorder in the absence of other recent pathology. If other pathology is present, a higher score of 5 on the SSSQ or 9 on the SFQ is suggestive of personality disturbance.

The level of agreement between social function and personality severity status in all three studies shows that personality status is the main driver of social dysfunction and a high score of such dysfunction in the absence of other explanations should make the clinician conscious of possible personality disturbance.The higher association shown in the Nottingham Study can be explained by the assessments at 12 and 30 years being carried out at a time determined by the original randomized trial, so these were not carried out at a time of clinical disturbance as in the trials of self‐harm and health anxiety. This suggests that the use of the SFQ and SSFQ as possible indicators of personality dysfunction might be most appropriate in epidemiological studies when the times of assessment are essentially chosen at random.

## CONFLICT OF INTERESTS

HT, MY and MC have no conflicts of interest to declare. PT was the originator of the SFQ.

## Appendix

Please look at the statements below and tick the reply that comes closest to how you have been generally.

**Table:**

1. I complete my tasks at work and home satisfactorily.	Most of the time Quite often Sometimes Not at all	☐ 0 ☐ 1 ☐ 2 ☐ 3
2. I find my tasks at work and at home very stressful.	Most of the time Quite often Sometimes Not at all	☐ 3 ☐ 2 ☐ 1 ☐ 0
3. I have difficulties in getting and keeping close relationships.	Severe difficulties Some problems Occasional problems No problems at all	☐ 3 ☐ 2 ☐ 1 ☐ 0
4. I feel lonely and isolated from other people.	Almost all the time Much of the time Not usually Not at all	☐ 3 ☐ 2 ☐ 1 ☐ 0
5. I enjoy my spare time.	Very much Sometimes Not often Not at all	☐ 0 ☐ 1 ☐ 2 ☐ 3
